# Non-antibiotic treatment of acute urinary tract infection in primary care: a qualitative study

**DOI:** 10.3399/BJGP.2021.0603

**Published:** 2022-03-22

**Authors:** Oghenekome A Gbinigie, Sarah Tonkin-Crine, Christoper C Butler, Carl J Heneghan, Anne-Marie Boylan

**Affiliations:** Nuffield Department of Primary Care Health Sciences, University of Oxford, Oxford.; Nuffield Department of Primary Care Health Sciences, University of Oxford; National Institute for Health Research Health Protection Research Unit in Healthcare Associated Infections and Antimicrobial Resistance, University of Oxford, Oxford.; Nuffield Department of Primary Care Health Sciences, University of Oxford, Oxford.; Nuffield Department of Primary Care Health Sciences, University of Oxford, Oxford.; Nuffield Department of Primary Care Health Sciences, University of Oxford, Oxford.

**Keywords:** delayed antibiotics, general practice, primary health care, qualitative research, urinary tract infections, vaccinium macrocarpon

## Abstract

**Background:**

The views of women with acute, uncomplicated urinary tract infection (auUTI) on the acceptability of non-antibiotic treatment options are poorly understood.

**Aim:**

To establish women’s thoughts on and experience of non-antibiotic treatment for auUTIs.

**Design and setting:**

Qualitative interview study with primary care patients in Oxfordshire, UK, embedded within the Cranberry for Urinary Tract Infection (CUTI) feasibility trial.

**Method:**

One-to-one, semi-structured interviews were conducted between August 2019 and January 2020 with some CUTI trial participants and some patients who were not part of the CUTI trial who had experienced at least one urinary tract infection (UTI) in the preceding 12 months in Oxfordshire, UK. Interviews were analysed using thematic analysis.

**Results:**

In total, 26 interviews were conducted and analysed. Women expected to receive an immediate antibiotic for their UTI but were aware of the potential harms of this approach. They were keen to find a non-antibiotic, ‘natural’ alternative that could effectively manage their symptoms. In certain situations (early illness, milder illness, and with no important upcoming engagements), women indicated they would be prepared to postpone antibiotic treatment by up to 3 days, especially if offered an interim non-antibiotic option with perceived therapeutic potential.

**Conclusion:**

Many women with auUTIs are open to trying non-antibiotic treatments first in certain situations. There is scope for more dialogue between primary care clinicians and patients with auUTI around delaying antibiotic treatment and using non-antibiotic options initially, which could reduce antibiotic consumption for this common infection.

## INTRODUCTION

As antibiotic resistance continues to rise,^1^ there has been growing interest in non-antibiotic treatments to manage common bacterial infections, such as acute, uncomplicated urinary tract infection (auUTI). AuUTIs are commonly managed in general practice,^2^ and are almost always treated with immediate antibiotics.^3^ However, many UTIs are self-limiting, and there is potential to avoid antibiotics.

Trials of non-steroidal anti-inflammatory drugs (NSAIDs)^4^^–^7 and herbal treatments^8^^,^^9^ for auUTI treatment have typically resulted in reduced antibiotic consumption but worse symptom control. Many trials of cranberry extract for UTI prevention have been conducted, with promising results.^10^^–^13 However, many have suffered methodological problems, such as high participant drop-out attributed to difficulty drinking large volumes of cranberry juice over extended periods.^11^ A systematic review found limited evidence for or against using cranberry extract to treat auUTIs.^14^ Despite this, up to 27% of women report consuming cranberry products for auUTI treatment and around 17% use cystitis sachets to help manage auUTIs,^15^ despite an absence of randomised trial evidence.^16^

In addition to establishing the efficacy of non-antibiotic treatments through clinical trials, it is critical to understand women’s thoughts on and experiences of using them for symptoms of auUTI. Such qualitative exploration provides an understanding of whether and how women might engage with such treatments if they were shown to be clinically effective and introduced into routine clinical practice. Previous qualitative research has established that a delayed antibiotic strategy may be acceptable to some women.^17^^,^^18^ However, limited studies have focused on exploring the acceptability of non-antibiotic treatments as part of a delayed antibiotic strategy for auUTI symptom management.^19^

The aim of this study was to explore women’s views on treating auUTIs with non-antibiotic treatments.

## METHOD

### Context and recruitment

Interviews were embedded within the Cranberry for Urinary Tract Infection (CUTI) feasibility trial, an open-label, randomised feasibility trial of the use of cranberry extract in treating symptoms of auUTI in primary care.^14^ In the CUTI trial, patients with auUTI presenting to participating general practices in Oxfordshire were randomly assigned to one of three groups:

- immediate antibiotic prescription;
- immediate antibiotic prescription and immediate cranberry capsules; or
- immediate cranberry capsules and a delayed antibiotic prescription in case symptoms worsened or did not improve within 3–5 days.

**Table:** How this fits in

To the authors’ knowledge, interview studies have not explored women’s views on using non-antibiotic treatments as a way of managing symptoms of acute, uncomplicated UTI. While women generally perceive antibiotics to be an effective and reliable treatment, they are aware of the potential harms associated with antibiotic consumption, frequently mentioning fears of becoming ‘immune’ to their effects. This study found that many women view non-antibiotic treatments, such as cranberry extract and cystitis sachets, positively, and are willing to try them in certain situations (for example, for early symptoms, milder symptoms, and when they do not have important upcoming engagements). There is scope for healthcare professionals to have more discussions with women about considering a delayed antibiotic strategy; offering a non-antibiotic treatment in the interim may make this approach more acceptable to women.

CUTI trial methods and results have been published in full elsewhere.^20^^,^^21^

Semi-structured interviews^22^ were conducted with a sample of CUTI trial participants and non-CUTI trial patients who had experienced at least one auUTI in the preceding 12 months. The non-CUTI trial patients were identified through an electronic search of women aged ≥18 years with an auUTI in the past 12 months conducted at a general practice in Oxfordshire, outwith the CUTI trial. This practice was chosen to facilitate maximum-variation sampling: it was in an area of higher deprivation and with more ethnic diversity compared with CUTI trial practices. Fully informed, written consent was obtained from each participant prior to being interviewed.

### Participants

The aim was to conduct 20–30 interviews with women aged ≥18 years who had experienced an auUTI in the preceding 12 months,^23^ with the final number determined by data saturation.^24^ Immunosuppressed women, women with underlying urological abnormalities, and women receiving palliative care were excluded as such women are more likely to experience complicated UTIs and require immediate antibiotics. The authors hoped to employ a purposive maximum-variation sampling strategy^25^ with regard to age, ethnicity, and whether or not women were CUTI trial participants.

### Data collection

A narrative, semi-structured interview guide^22^ was used to explore participants’ experiences (Supplementary Appendix S1). The topic guide was developed by the lead author through review of literature on non-antibiotic treatments of UTIs, in consultation with one other author, and was reviewed by patient and public involvement (PPI) contributors. Patients with a UTI were encouraged to tell their story about their most recent UTI from when they first suspected they had a UTI to the end of the illness episode. Additional questions elicited further details on help-seeking behaviour, self-care strategies, thoughts on non-antibiotic treatments, and experience of taking part in the CUTI trial, where relevant. All interviews were conducted by the lead author, audio-recorded, and professionally transcribed verbatim.

### Philosophical approach

An interpretivist approach was taken,^26^ recognising that a person’s beliefs about UTI and non-antibiotic treatments are dependent on their prior experiences, their context, and interactions they have had. These beliefs are also changeable in light of new experiences, contexts, and interactions (for example, moving to a different country with a different healthcare system).

### Data analysis

A thematic analysis was conducted with analysis and data collection performed concurrently. Thematic analysis involves constructing and analysing patterns (or themes) within data.^27^ The lead author read transcripts and listened to audio-recordings several times to aid familiarisation, allowing immersion in the data. NVivo (version 12) software was used to organise the data and facilitate coding. The lead author grouped codes relating to similar phenomena into categories, and, in discussion with two other authors, subsequently generated themes and subthemes to describe the data through an iterative process. Once the thematic structure was finalised, theme labels were refined to comprehensively describe the data within, and supporting quotes chosen to illustrate the themes and subthemes.

### PPI

Four PPI contributors were involved with the CUTI trial and interview studies from the outset. They reviewed all public-facing documents (such as the participant information leaflet). The developing analysis was shared with PPI contributors to seek their thoughts on the findings, which were incorporated into the analyses.

## RESULTS

In total, 27 interviews were conducted with CUTI trial participants (*n* = 14) and non-trial UTI patients (*n* = 13) between August 2019 and January 2020. One interview with a non-trial UTI patient was not analysed as the participant met an exclusion criterion (immunosuppressed). Interviews ranged from 28–72 minutes (mean 54 minutes). Participant characteristics are described in Table 1.

**Table 1.** Characteristics of interview participants

	**CUTI trial group 1 participants**	**CUTI trial group 2 participants**	**CUTI trial group 3 participants**	**Non-trial UTI patients**
Number	6	5	3	12 ^a^

ID numbers	T2, T7, T10, T11, T12, T13	T4, T5, T6, T8, T14	T1, T3, T9	NT2-NT13

Age, years, mean (range)	67 (32–81)	69 (60–77)	44 (23–57)	45 (18–76)

Ethnic group, *n*	White, English = 6	White, English = 3	White, English = 2	White, English = 2
		White, British = 1	White, British = 1	White, British = 4
		White, Welsh = 1		White, Spanish = 2
				White, Lithuanian = 1
				White, Bulgarian = 1
				White, European = 1
				White, Australian = 1

Marital status, *n*	Married = 3	Married = 4	Single, never married = 1	Single, never married = 5
	Widowed = 2	Widowed = 1	Married = 1	Domestic Partnership = 2
	Divorced = 1		Separated = 1	Married = 4
				Widowed = 1

Employment status, *n*	Retired = 4	Retired = 4	Employed for wages = 1	Retired = 3
	Employed for wages = 1	Self-employed = 1	Out of work and looking for work = 1	Employed for wages = 7
	Self-employed = 1		Self-employed = 1	Unable to work, medically = 1
				Student = 1

Highest level of school/degree, *n*	GCSE level = 1	GCSE level = 2	A-level = 1	GCSE level = 1
	A-level = 1	College qualification or equivalent = 2	College qualification or equivalent = 1	A-level = 1
	College qualification or equivalent = 2	University Bachelor’s degree = 1	Vocational training = 1	College qualification or equivalent = 1
	Vocational training = 1			University Bachelor’s degree = 4
	University Bachelor’s degree = 1			Master’s degree = 3
				Doctorate degree = 2

a *The interview with NT1 was not analysed as the participant met an exclusion criterion (immunosuppressed). CUTI = Cranberry for Urinary Tract Infection. GCSE = General Certificate of Secondary Education.*

The three themes presented here demonstrate women’s thoughts on and experiences of UTI management, including: self-care; treatments (immediate/delayed antibiotics and non-antibiotic options, within and outwith the CUTI trial); and help-seeking behaviour.

### Theme 1: treatments, cures, and symptom control

Women often spoke of finding a ‘cure’ for their UTI, and for many women antibiotics represented a cure: most reported finding antibiotics an effective treatment for UTIs, which they perceived worked quickly. Interviewees often expressed a tension between finding antibiotics effective while wanting to avoid taking them if possible. A recurring sentiment expressed was fear of becoming ‘immune’ to antibiotics if they took them frequently:

*‘You don’t sort of want to, you know, take too many of them* [antibiotics] *and then like the next episode you have, you cannot sort of treat it well, well enough, you know.’*

(Non-trial participant [NT]8, aged 31 years)

For other women, typically women who had experienced recurrent UTIs or protracted UTI symptoms, a cure instead implied a permanent end to their UTIs, which went above and beyond their perceived capabilities of an antibiotic:

Lead author: *‘… when you then contacted your GP practice … what were you sort of hoping for?’*

NT6: *‘I think I was … hoping for a cure but knowing that perhaps I would only get antibiotics.’* (NT6, 62 years)

Over-the-counter (OTC) remedies, such as cystitis sachets and cranberry products, were generally seen as more ‘natural’ and more easily accessible than antibiotics, but less reliably effective. Some women used them as holding measures to provide symptom relief when it was not possible to get a GP appointment. However, other women used them as a UTI treatment in their own right, particularly with milder symptoms, in the earlier stages of their illness, and if they did not have an important upcoming engagement (for example, going on holiday).

*‘They’re* [cystitis sachets] *quite effective if you, if you get it early but once an infection takes hold, they’re, they’re not very, in my, from my experience, not very effective.’*

(NT12, 46 years)

Women frequently reported that they would increase their fluid intake as an early part of their UTI management. Similarly to OTC remedies, increasing fluid intake was viewed by some as a holding measure. However, while women usually discontinued OTC measures when starting antibiotic treatment, women often continued increased fluid intake alongside taking antibiotic treatment, perceiving it as a treatment adjunct and a way to ‘flush out’ the infection. Some women felt that a mild UTI could even be treated through increased fluid intake alone:

*‘If it’s* [the UTI] *really mild … you can flush it out with water … ‘*

(Trial participant [T]10, 77 years)

Cranberry juice was commonly reported in this context. Many women perceived that cranberry juice might have specific therapeutic properties over and above other fluids:

*‘I’ve heard cranberry mentioned so much over the past years, going back from my own GP who is saying, you know, “Drink, drink cranberry juice and whatever.” So it’s always been in the loop … ‘*

(T7, 81 years)

Few women were aware of, or had tried, cranberry in capsule/tablet form, prior to taking part in the CUTI trial/interview study. Women usually felt that cranberry capsules/tablets would be preferable to consuming cranberry juice, because of concerns about the taste and sugar content of juice formulations. Those women who had used cranberry tablets (outside of the CUTI trial) usually reported using them as a means of preventing UTI, rather than as a way of managing an acute UTI:

*‘I’ve been taking the cranberry tablets just sort of daily.’*

(NT13, 32 years)

Most women did not naturally link analgesia (such as paracetamol and ibuprofen) with treating a UTI. Women who reported taking analgesia saw it as a means of alleviating certain symptoms (such as abdominal pain), but not as a way of treating their UTI:

*‘It* [taking analgesia] *wasn’t something I really thought or associated really … I was thinking really more about actually making what was causing it better rather than actually taking the painkillers to mask it.*’

(T3, 51 years)

### Theme 2: functional and formulaic — UTI consultations in general practice and the role of the healthcare practitioner

Women typically contacted their GP when they perceived that their symptoms were severe and/or inconveniencing. Physical evidence, namely haematuria, was seen as confirmation that symptoms warranted attention and a legitimate reason for seeking medical attention:

*‘If I see blood in my urine that’s like the sign I need to ring a GP … it’s not just in my mind … something is going on.’*

(NT8, 31 years)

On contacting their GP, women hoped to be seen quickly by a healthcare practitioner and expected an immediate antibiotic prescription; consultations appeared to be set up to meet this expectation:

*‘I rang the doctor’s appointment and asked them if I could come and expecting them to maybe just give me some antibiotics … because before when I’ve gone in, I’ve always had antibiotics …’*

(T1, 23 years)

Women described quick and focused consultations, and sometimes sensed that healthcare practitioners seemed to be following an algorithm, for which the outcome was usually an antibiotic prescription:

*‘They will just say, infection yes or no; antibiotics, yes or no.’*

(NT13, 32 years)

*‘They checked the urine and they said, “You have a UTI; we’ll give you antibiotics.”’*

(NT7, 18 years)

Outside of the CUTI trial, discussions about non-antibiotic treatments with healthcare practitioners were unusual. Women described that healthcare professionals tended to express negative sentiments (stating that non-antibiotic options did not work) or neutral feelings (stating that non-antibiotic options were unlikely to help, but unlikely to do harm):

*‘I said … “I can recover without antibiotics?” but he said, “No”, so. So was very clear. He said, “Antibiotics or nothing, but nothing, make sure you are going to get worse.” He didn’t mention about there is another method.’*

(NT9, 40 years)

*‘There’s certain doctors that I think probably don’t really talk about over-the-counter stuff and if you mention it they’ll be, like, dubious.’*

(NT5, 27 years)

Some women also suggested that different healthcare professionals provided conflicting advice about OTC treatments. Despite this, interviewees usually stated that their view of OTC remedies would be influenced by the views and recommendations of their healthcare practitioner, and the relationship they share:

*‘I trust my doctor if I was talking to him, if he advised something, I would take it because I trust him. I know him very well and he knows me.’*

(T10, 77 years)

*‘I don’t feel knowledgeable enough to go and pick something up and feel like, yes this is going to work. I suppose unless somebody in the health profession had recommended me to do so.’*

(T12, 32 years)

### Theme 3: changing the treatment paradigm

Women tended not to report that a delayed antibiotic strategy had been used in the management of their UTIs, outside of the CUTI trial. Some women considered that the delayed antibiotic approach provided a welcome opportunity to avoid consuming antibiotics for an acute UTI. However, women weighed this potential benefit against other factors, such as the severity of their symptoms and the timing of their presentation to general practice. A few women, typically those who had previously experienced an upper UTI, also factored in the potential risk of developing a complicated UTI if antibiotic treatment was delayed:

*‘I might be a bit grumpy about it. I think it would depend how awful I felt … ‘*

(NT3, 65 years)

Many women felt that a delay of 3–5 days (as suggested in the CUTI trial) was too long; a shorter delay of 2–3 days was generally considered more acceptable. Women expressed that having contacted their GP it was important for them to receive something by way of treatment in the interim:

*‘I’m not sure why you’d just delay antibiotics without doing anything because the whole reason for going to your doctor is that you’ve reached a decision, a big decision that you want to go the doctor to get it sorted out and the fact that the doctor says we’ll just wait another couple of days, you might already have waited three, four, five days before you made that decision to go to the doctor … ‘*

(T14, 60 years)

Women in the CUTI trial were randomly assigned to one of three groups. Group 1 of the trial (immediate antibiotics alone) aligned with what most women had originally hoped for on contacting their GP, and was therefore the preference for some women. This was particularly the case for women who were experiencing perceived severe symptoms, or who had already delayed seeking medical attention.

Group 2 (immediate antibiotics and immediate cranberry capsules) was seen by some as the ‘best of both worlds,’ allowing them to experience the benefit of taking an antibiotic along with any potential additional beneficial effect of cranberry:

*‘Probably the safest one would be the immediate antibiotics and the cranberry capsules because you get both basically, so that’s like a, you know, a full force attack … ’*

(NT8, 31 years)

Women assigned to this group compared their UTI experience within the trial with their previous UTI experiences that had been managed with antibiotics alone, and often reflected that they perceived an additive benefit to taking cranberry alongside antibiotics:

*‘Between the two* [antibiotics and cranberry capsules] *, it cleared it up very quickly … it was really, really helpful … ‘*

(T8, 77 years)

*‘I took the cranberry tablets alongside* [antibiotics] *and I thought the symptoms had gone but within a couple of days, I felt they were coming back again.* [I] *Took some cranberry tablets … I took another two days’ worth … I think that probably if I hadn’t had the cranberry tablets I would have had to go back to the GP for more antibiotics.’*

(T5, 60 years)

However, other women primarily saw the utility of cranberry as a way of avoiding antibiotics. Group 3 (immediate cranberry and a delayed antibiotic prescription in case symptoms worsened or did not improve within 3–5 days) was therefore the preferred group for women expressing this view. These women were keen to establish whether cranberry extract would help them personally and felt that combining cranberry with antibiotics would make this more difficult to ascertain. Women felt reassured that they would receive back-up antibiotics, which one woman described as a ‘parachute’, giving confidence to try a new intervention.

While some women in group 3 of the trial were able to avoid taking antibiotics,^21^ of the women who were interviewed in this group all ended up taking their delayed antibiotic prescription. However, some women interviewed in this group suggested that cranberry had some effect, albeit not as potent as antibiotics, and seemed to prevent symptom deterioration:

*‘I felt that actually just taking the tablet, the cranberry tablets it was just like, it didn’t get rid of it, but it helped, I think it prevented it from getting any worse. So, the actual like burning feeling and needing to go for a wee, I felt like it was a steady level.’*

(T1, 23 years)

## DISCUSSION

### Summary

Acute UTI symptoms can be severe and disruptive to women’s lives. Antibiotics were usually perceived as a reliable treatment, or indeed a cure. However, responders were also aware of potential harms associated with antibiotics, such as becoming ‘immune’ to their effects. Non-antibiotic measures were variably used as UTI treatments, treatment adjuncts, holding measures, or for symptom relief. Non-antibiotic measures were perceived as more natural but less potent than antibiotics, with better results and greater acceptability if used earlier in the illness course, with milder symptoms, and when patients had no important upcoming engagements.

Women were willing to consider a delayed antibiotic approach, but this option was not usually offered in GP consultations. The perceived binary choice between antibiotics or no antibiotics did not appear to leave much room for wider discussions. The acceptability of the delayed antibiotic approach was improved by offering an interim non-antibiotic treatment with perceived therapeutic potential.

### Strengths and limitations

Interviews were conducted with patients with UTIs within and outwith the CUTI trial. Interviews were conducted with non-CUTI trial participants to increase the chance of capturing the views and experiences of women who might be less amenable to trying non-antibiotic treatments. However, it is possible that women who were interested in non-antibiotic treatments for UTI were more likely to respond positively to the interview invite.

Interviews were conducted with women of a range of ages and from a range of backgrounds. A diverse sample was attempted by recruiting non-CUTI trial participants from a general practice with more ethnic diversity compared with the CUTI trial practices. However, despite best efforts, there was limited ethnic diversity in the sample. The results may therefore be less reflective of the experiences of women with UTIs from minority ethnic groups. Furthermore, electronic searches may have missed some potentially eligible patients.

All interviews were conducted by the chief investigator of the CUTI trial (the lead author), which might have influenced the participants’ responses. To minimise the potential for this, participants were reminded before the interviews that they were the experts in their experiences and that the interviewer was keen to hear all views.

Finally, ethical approval to interview women who declined to take part in the CUTI trial was not received. Interviewing CUTI trial decliners may have provided additional, useful insights.

### Comparison with existing literature

The findings of the present study suggest that women would consider delaying antibiotics in certain situations, such as with earlier and milder UTI symptoms. This is in keeping with previous research that explored GP and patient experiences of delayed prescribing for UTIs.^18^ The authors concluded that the decision to delay antibiotics should depend on whether self-management strategies have already been tried before seeing the GP and symptom severity.

Leydon *et al*^17^ also explored women’s thoughts on and experiences of delayed antibiotics for UTIs and found that women would be prepared to delay antibiotics if their symptoms were not severe. The authors also suggest that GPs should be mindful that women may not always want immediate antibiotics. The present study builds on these findings and provides additional unique insights that suggest that the acceptability of a delayed antibiotic strategy for UTI may be increased by receiving a non-antibiotic alternative treatment in the interim.

Interviews with women taking part in a trial of immediate antibiotics versus immediate ibuprofen for auUTI in Germany showed they perceived that it was safe to take part because UTI is *‘not a serious condition’*.^19^ Indeed, four of the five trial decliners interviewed had refused trial participation on the basis that they wished to avoid immediate antibiotics. This contrasts with the findings of the present study, in which women reported significant, disruptive symptoms associated with UTI. Furthermore, the commonest reason for women declining to take part in the CUTI feasibility trial was to avoid being assigned to the delayed antibiotics group.^21^ This difference may represent cultural differences in the way that UTIs and antibiotics are perceived. Of note, outpatient antimicrobial prescription in Germany is lower than in the UK.^28^

A number of studies have evaluated the use of ibuprofen as an alternative treatment for acute UTIs.^4^^–^7 However, women interviewed in the present study did not perceive analgesia as a UTI treatment but rather a means of alleviating pain. This is in keeping with a questionnaire study of women with UTI, which found that most women take antibiotics because they want to *‘combat bacteria’*.^29^

The need to ‘combat bacteria’ may also be prevalent among healthcare practitioners. In an interview study exploring GPs’ experience of delayed antibiotic prescribing for a UTI, GPs felt that a firm UTI diagnosis warranted antibiotics.^18^ They might consider a delayed antibiotic approach for equivocal UTI symptoms but were more used to applying the delayed antibiotic approach in the context of acute respiratory tract infections.^18^ Urine samples sent for culture are the commonest specimens sent to microbiological laboratories.^30^ Healthcare practitioners routinely receiving urine culture results that show bacterial growth may serve to reinforce immediate antibiotic prescribing behaviour. As throat swabs/sputum cultures are not routinely sent to laboratories for respiratory infections in primary care,^31^^,^^32^ the same positive reinforcement of antibiotic prescribing behaviour may not be present for acute respiratory tract illnesses.

### Implications for research and practice

Women with auUTIs are amenable to trying certain non-antibiotic treatments with advice to delay antibiotics for a short period, in some situations. National Institute for Health and Care Excellence guidance suggests that a delayed antibiotic prescription can be considered for women with auUTIs, taking into consideration various factors such as symptom severity and patient preference.^33^ The present study, in keeping with previous studies,^17^^,^^18^ suggests that this does not happen often. There is therefore scope for clinicians to have more discussions with women about a delayed antibiotic approach, particularly if women have presented earlier in their illness, have milder symptoms, and do not have pressing upcoming engagements. Such consultations should be sensitively conducted, recognising that the patient is the expert in their own symptoms and is best placed to make an appropriate decision.

Clinicians should also bear in mind that, if a patient rejects a delayed antibiotic approach on one occasion, this does not necessarily mean that they will reject it in future; often, the decision is dependent on their experience of the UTI episode in question, rather than being a fixed preference. These discussions may lengthen a consultation, but they may have the potential to reduce antibiotic consumption and empower women to take control of their acute UTI management. While a delayed antibiotic approach will not prevent all women from consuming antibiotics, given that auUTIs are common and are almost always managed with an immediate antibiotic prescription,^3^ there is scope to meaningfully reduce antibiotic consumption for auUTIs through taking this approach.

A delayed antibiotic prescription may be more acceptable to women if they receive an interim, non-antibiotic treatment that they perceive to be potentially effective. The results of the CUTI feasibility trial are suggestive of possible, preliminary evidence of an effect of cranberry extract on reducing antibiotic consumption for acute UTI.^21^ Adequately powered clinical trials are needed to definitively establish whether non-antibiotic treatments like cranberry and cystitis sachets are safe and effective treatments, and to better define when delayed antibiotics are suitable. Ideally, these trials should include a qualitative evaluation to better understand whether/when/how women might find non-antibiotic treatments acceptable when integrated into auUTI management. Such trials and interviews should incorporate the views of people from diverse ethnicities, for example, by engaging community-bridging researchers who speak a variety of languages.^34^

## References

[1] World Health Organization (2014). *Antimicrobial resistance. Global report on surveillance 2014*.

[2] Butler CC, Hillier S, Roberts Z (2006). Antibiotic-resistant infections in primary care are symptomatic for longer and increase workload: outcomes for patients with E. coli UTIs. Br J Gen Pract.

[3] Little P, Merriman R, Turner S (2010). Presentation, pattern, and natural course of severe symptoms, and role of antibiotics and antibiotic resistance among patients presenting with suspected uncomplicated urinary tract infection in primary care: observational study. BMJ.

[4] Gágyor I, Bleidorn J, Kochen MM (2015). Ibuprofen versus fosfomycin for uncomplicated urinary tract infection in women: randomised controlled trial. BMJ.

[5] Bleidorn J, Gágyor I, Kochen MM (2010). Symptomatic treatment (ibuprofen) or antibiotics (ciprofloxacin) for uncomplicated urinary tract infection? — results of a randomized controlled pilot trial. BMC Med.

[6] Vik I, Bollestad M, Grude N (2018). Ibuprofen versus pivmecillinam for uncomplicated urinary tract infection in women — a double-blind, randomized non-inferiority trial. PLoS Med.

[7] Kronenberg A, Bütikofer L, Odutayo A (2017). Symptomatic treatment of uncomplicated lower urinary tract infections in the ambulatory setting: randomised, double blind trial. BMJ.

[8] Wagenlehner FM, Abramov-Sommariva D, Höller M (2018). Non-antibiotic herbal therapy (BNO 1045) versus antibiotic therapy (Fosfomycin Trometamol) for the treatment of acute lower uncomplicated urinary tract infections in women: a double-blind, parallel-group, randomized, multicentre, non-inferiority phase III trial. J Urol Int.

[9] Moore M, Trill J, Simpson C (2019). Uva-ursi extract and ibuprofen as alternative treatments for uncomplicated urinary tract infection in women (ATAFUTI): a factorial randomized trial. Clin Microbiol Infect.

[10] Wang C-H, Fang C-C, Chen N-C (2012). Cranberry-containing products for prevention of urinary tract infections in susceptible populations: a systematic review and meta-analysis of randomized controlled trials. Arch Intern Med.

[11] Jepson RG, Williams G, Craig JC (2012). Cranberries for preventing urinary tract infections. Cochrane Database Syst Rev.

[12] Fu Z, Liska D, Talan D, Chung M (2017). Cranberry reduces the risk of urinary tract infection recurrence in otherwise healthy women: a systematic review and meta-analysis. J Nutr.

[13] Luís Â, Domingues F, Pereira L (2017). Can cranberries contribute to reduce the incidence of urinary tract infections? A systematic review with meta-analysis and trial sequential analysis of clinical trials. J Urol.

[14] Gbinigie OA, Spencer EA, Heneghan CJ (2021). Cranberry extract for symptoms of acute, uncomplicated urinary tract infection: a systematic review. Antibiotics.

[15] Butler CC, Hawking MK, Quigley A, McNulty CA (2015). Incidence, severity, help seeking, and management of uncomplicated urinary tract infection: a population-based survey. Br J Gen Pract.

[16] O’Kane DB, Dave SK, Gore N (2016). Urinary alkalisation for uncomplicated urinary tract infection in women. Cochrane Database Syst Rev.

[17] Leydon G, Turner S, Smith H, Little P (2010). Women’s views about management and cause of urinary tract infection: qualitative interview study. BMJ.

[18] Duane S, Beatty P, Murphy AW, Vellinga A (2016). Exploring experiences of delayed prescribing and symptomatic treatment for urinary tract infections among general practitioners and patients in ambulatory care: a qualitative study. Antibiotics.

[19] Bleidorn J, Bucak S, Gágyor I (2015). Why do — or don’t — patients with urinary tract infection participate in a clinical trial? A qualitative study in German family medicine.. Ger Med Sci.

[20] Gbinigie O, Allen J, Boylan A-M (2019). Does cranberry extract reduce antibiotic use for symptoms of acute uncomplicated urinary tract infections (CUTI)? Protocol for a feasibility study. Trials.

[21] Gbinigie O, Allen J, Williams N (2021). Does cranberry extract reduce antibiotic use for symptoms of acute uncomplicated urinary tract infections (CUTI)? A feasibility randomised trial. BMJ Open.

[22] Smith JA, Harré R, Van Langenhove L (1995). Rethinking methods in psychology.

[23] Baker SE, Edwards R (2012). How many qualitative interviews is enough? Expert voices and early career reflections on sampling and cases in qualitative research.

[24] Strauss A, Corbin J (1998). Basics of qualitative research: techniques and procedures for developing grounded theory.

[25] Coyne IT (1997). Sampling in qualitative research. Purposeful and theoretical sampling; merging or clear boundaries?. J Adv Nurs.

[26] Smith JK (2004). Quantitative versus interpretive: the problem of conducting social inquiry. New Dir Program Eval.

[27] Braun V, Clarke V (2006). Using thematic analysis in psychology. Qual Res Psychol.

[28] Van De Sande-Bruinsma N, Grundmann H, Verloo D (2008). Antimicrobial drug use and resistance in Europe. Emerg Infect Dis.

[29] Willems CS, van den Broek D’Obrenan J, Numans ME (2014). Cystitis: antibiotic prescribing, consultation, attitudes and opinions. Fam Pract.

[30] Morgan M, McKenzie H (1993). Controversies in the laboratory diagnosis of community-acquired urinary tract infection. Eur J Clin Microbiol Infect Dis.

[31] National Institute for Health and Care Excellence (2021). Sore throat — acute: how do I diagnose the cause of a sore throat?. https://cks.nice.org.uk/topics/sore-throat-acute/diagnosis/diagnosing-the-cause/.

[32] National Institute for Health and Care Excellence (2021). Chest infections — adult: how should I assess an adult with a suspected chest infection?. https://cks.nice.org.uk/topics/chest-infections-adult/diagnosis/assessment/.

[33] National Institute for Health and Care Excellence (2018). Urinary tract infection (lower): antimicrobial prescribing NG109.

[34] Prinjha S, Miah N, Ali E, Farmer A (2020). Including ‘seldom heard’views in research: opportunities, challenges and recommendations from focus groups with British South Asian people with type 2 diabetes. BMC Med Res Methodol.

